# Use of diabetes technology in children

**DOI:** 10.1007/s00125-024-06218-0

**Published:** 2024-07-12

**Authors:** Melissa J. Schoelwer, Mark D. DeBoer, Marc D. Breton

**Affiliations:** 1 Department of Pediatrics, University of Virginia, Charlottesville, VA USA; 2 Center for Diabetes Technology, University of Virginia, Charlottesville, VA USA

**Keywords:** Automated insulin delivery, Continuous glucose monitoring, Diabetes technology, Medical technology, Paediatric care, Review, Type 1 diabetes

## Abstract

Children with type 1 diabetes and their caregivers face numerous challenges navigating the unpredictability of this complex disease. Although the burden of managing diabetes remains significant, new technology has eased some of the load and allowed children with type 1 diabetes to achieve tighter glycaemic management without fear of excess hypoglycaemia. Continuous glucose monitor use alone improves outcomes and is considered standard of care for paediatric type 1 diabetes management. Similarly, automated insulin delivery (AID) systems have proven to be safe and effective for children as young as 2 years of age. AID use improves not only blood glucose levels but also quality of life for children with type 1 diabetes and their caregivers and should be strongly considered for all youth with type 1 diabetes if available and affordable. Here, we review key data on the use of diabetes technology in the paediatric population and discuss management issues unique to children and adolescents.

## Introduction

Diabetes is one of the most common chronic health conditions seen in children, and recent studies show that the incidence of both type 1 and type 2 diabetes is increasing in childhood [1, 2]. The need for therapeutic and technological advances to improve glycaemic outcomes in the paediatric population cannot be understated, as duration of diabetes and younger age at diagnosis are linked to excess mortality and increased risk for CVD later in life [3].

Conventional therapy for children with type 1 diabetes consists of either multiple daily insulin injections (MDI) or continuous insulin infusion via an insulin pump, with the dose of insulin for meals calculated based on the glucose level prior to eating and the carbohydrate content of the food to be consumed. The management of type 1 diabetes is quite difficult in growing children as their insulin requirements change continuously, often increasing as they grow. Insulin sensitivity is impacted by growth hormone and cortisol release [4]. Hormones fluctuate by time of day and vary by age, creating unique patterns of insulin needs throughout childhood. Puberty, a time of significant insulin resistance, is particularly challenging as insulin needs rise rapidly. Consequently, frequent adjustments to insulin doses, including in between regular visits to a paediatric endocrinologist every 3 months, are necessary to achieve adequate glycaemic management in the paediatric type 1 diabetes population.

Over the past two decades, diabetes management has been revolutionised by the use of technology, including continuous glucose monitors (CGMs) and automated insulin delivery (AID) systems. Its use is rapidly increasing in children in many areas of the world and the safety and efficacy of AID is now well documented [5, 6]. CGM use alone improves metabolic outcomes, even when not combined with a pump or AID system, and AID clearly outperforms traditional open-loop insulin pumps in children with type 1 diabetes [7, 8]. In this review, we will provide an update on technology used in the paediatric diabetes population, discussing the impact of technology on both glycaemic and patient-reported outcomes and the application of technology use in the real world. We will also review barriers that remain to increased technology uptake and discuss where research in this field is headed.

## Paediatric glycaemic goals

While it is well known that repeated, severe hypoglycaemia is harmful to the developing brain, the negative impact of both acute and chronic hyperglycaemia on brain development and function in childhood has also recently become an area of concern [9]. Adults with diabetes are at increased risk of dementia and cognitive dysfunction, speculated to be related to changes in brain development in individuals with childhood-onset type 1 diabetes [10]. Recently, a longitudinal study confirmed lower brain volume and cognitive scores in children with type 1 diabetes compared with their matched peers without diabetes, the degree of which was associated with hyperglycaemia and glucose variability [11].

New technology has allowed children with type 1 diabetes to safely achieve tighter glycaemic management, and the glycaemic targets for children have shifted accordingly. Nearly two decades ago, HbA_1c_ goals as recommended by the ADA for children with type 1 diabetes were 58–69 mmol/mol (7.5–8.5%) for children <6 years of age, <64 mmol/mol (<8%) for children 6–12 years old and <58 mmol/mol (<7.5%) for adolescents [12]. In 2019, the recommendation was <58 mmol/mol (<7.5%) across all paediatric ages and today the goal of <53 mmol/mol (<7%) is recommended for most children by both the ADA and the International Society for Paediatric and Adolescent Diabetes [13, 14]. Importantly, that goal applies only to children who have access to advanced technologies and specialist diabetes care. Despite the decreasing HbA_1c_ target over time, studies consistently show that, on average, the HbA_1c_ remains well above this goal in children, particularly in early adolescence, even as therapies have improved [15].

Although HbA_1c_ remains a gold standard marker of glucose management, CGM data allows for a more in-depth understanding of an individual’s metabolic excursions, giving important information on blood glucose variability as well as time spent in hypoglycaemia that is lacking with HbA_1c_ measurement alone. CGM consensus guidelines published in 2019 established recommended goals for CGM metrics across all ages including time in range (TIR; glucose levels 3.9–10 mmol/l [70–180 mg/dl]; goal >70%) and time below range (<3.9 mmol/l [<70 mg/dl]; goal <4%) [16]. However, given the many challenges of type 1 diabetes management in childhood, it is not surprising that children are less likely to meet these glycaemic targets as compared with adults [15, 17].

## Rising technology use

CGM use in paediatrics has rapidly expanded over the past 15 years as accuracy, availability and insurance coverage increased. Centres across the USA participating in the T1D Exchange Registry reported more than a tenfold increase in CGM use in the 2016–2018 cohort compared with the 2010–2012 cohort [15]. Data from the SWEET Registry, which includes centres in Europe, North America, Asia/Middle East and Australia, also revealed increasing CGM and pump use in children around the same time [8]. A recent population-based study from Australia reported a remarkable increase in CGM uptake from 5% to 79% following the introduction of universal subsidised CGM funding for people with type 1 diabetes under the age of 21 years [18].

AID use is similarly increasing in children with type 1 diabetes, although this technology is not as widespread as CGM and access is highly variable across the world. Multiple AID systems recently became commercially available in the USA and Europe, and uptake in the USA is increasing fairly rapidly based on early reports [19]. Interestingly, technology use, including pump use, is highly variable across clinical centres even within the same country [20].

## AID in school-aged children and adolescents

As paediatric AID research has moved from the inpatient setting to supervised hotel studies, to extended use at summer/winter camps and then prolonged home use, it has become very clear that AID systems are safe and effective for children with type 1 diabetes. It is beyond the scope of this review to include literature leading up to the commercialisation of AID systems. Data from paediatric pivotal studies of currently available AID systems (Table 1) show improved glycaemic outcomes with AID [21–28]. Of note, direct comparisons of systems cannot be made due to variable study design and differing CGMs used. Additionally, multiple open-source or ‘do-it-yourself’ systems are also available but are not covered in this review.

**Table 1:** AID pivotal studies in children and adolescents

Study detail	Medtronic 670G [21]	Tandem Control-IQ [23, 24]		Omnipod 5 [25]	Medtronic 780G [26]	iLet [27]	CamAPS FX [22, 28]	
Date	2016	2019	2020	2021	2022	2022	2018	2022
Study design	Single arm	RCT	RCT	Single arm	Single arm	RCT	RCT	RCT
Comparator	2 week run-in	CGM + pump	CGM + pump	2 week run-in	2 week run-in	Usual care (~30% on AID)	CGM + pump	CGM + pump
AID duration, months	3	6	4	3	3	3	4	6
No. of participants	124	168	101	240	157	326	86	133
Participant age, years	14–75	14–71	6–13	6–70	14–75	6 and older	6 and older	6–18
Baseline HbA_1c_, mmol/mol (%)	57	57	58 / 61	55	58	61 / 63	67 / 66	66 67
Baseline HbA_1c_^a^, %	7.4	7.4	7.5 / 7.7	7.2	7.5	7.7 / 7.9	8.3 / 8.2	8.2 / 8.3
ΔTIR, %	+5	+11	+12.4	+9	+5.7	+11	+10.8	+6.7
ΔHbA_1c_, %	−0.5	−0.33	−0.42	−0.38	−0.5	−0.5	−0.36	−0.32

^a^Where two HbA_1c_ values are given, the papers did not report a mean baseline HbA_1c_ value for the entire group, but rather listed baseline HbA_1c_ values separately for the closed-loop and control groups, respectively

Although AID use improves glycaemic outcomes and eases the burden of keeping up with increasing insulin needs as children grow and go through puberty, challenges in this age range persist. Current AID options are all hybrid closed-loop systems, meaning that the user is expected to announce meals and in most cases provide the carbohydrate content. One of the newest AID systems on the market allows for an estimation of carbohydrate intake (usual/more/less) but the need for an announcement of a meal remains [27]. Missed or late meal announcements are common in older children and adolescents and might occur more often in adolescents on AID as users learn that the system will compensate by increasing the insulin delivery in response to postprandial hyperglycaemia [29–31].

Children and adolescents who are already doing well prior to AID initiation tend to achieve the highest TIR on AID, while those who have worse glucose management at baseline exhibit the greatest improvement in TIR [32]. This highlights the important points that essentially all children benefit from AID and that the vast majority are candidates for AID use even if they traditionally would not have been thought of as a ‘good’ candidate for an open-loop pump in the past.

## AID in toddlers and preschoolers

Children between the ages of 2 and 5 years present unique challenges to diabetes management. Toddlers are often unpredictable when it comes to meal intake and activity. They have low insulin needs and can be exquisitely sensitive to small doses of insulin, limiting the ability of even a half-unit insulin pen or syringe to match their needs. Young children often have hypoglycaemia unawareness or might not be unable to communicate their symptoms of hypoglycaemia. Parents and caregivers of young children are understandably fearful of hypoglycaemia and sometimes contribute to excess hyperglycaemia by over-treating lows, under-dosing insulin or providing extra uncovered snacks when they might not be needed. This age group is entirely reliant on their parents and caregivers for their diabetes management, which often means better glycaemic control as compared with older children and adolescents due to infrequent missed meal boluses. However, this around-the-clock care can be quite burdensome to parents and greatly limits their childcare options [33].

Despite these many challenges, AID systems work well for most young children, even those with low total daily insulin needs, and there are now multiple commercially available AID options for children with type 1 diabetes down to age 2 years in the USA and 1 year in Europe. Table 2 summarises the pivotal study data for each system in this age group [34–37].

**Table 2:** AID pivotal studies in children under the age of 6 years

Study detail	Omnipod 5 [34]	Medtronic 670G [35]	CamAPS FX [36]	Tandem Control-IQ [37]
Date	2022	2022	2022	2023
Study design	Single arm	Single arm	RCT, crossover	RCT
Comparator	2 week run-in	2 week run-in	CGM + pump	CGM + pump / MDI
AID duration	13 weeks	12 weeks	16 weeks	13 weeks
No. of participants	80	46	74	102
Participant age, years	2–5.9	2–6	1–7	2–5.9
Baseline HbA_1c_, mmol/mol	57	64	56	58
Baseline HbA_1c_, %	7.4	8	7.3	7.5
ΔTIR, %	+10.9	+8.1	+8.7	+12.4
ΔHbA_1c_, %	−0.55	−0.5	−0.4	−0.42

## Patient-reported outcomes

The impact of diabetes technology on the lives of children with type 1 diabetes and their families extends well beyond improved glycaemic outcomes. Early CGM introduction following type 1 diabetes diagnosis in children is associated with reduced hypoglycaemia avoidance behaviours in parents [38]. Parents of children using AID report a reduction in the burden of diabetes, reduced fear of hypoglycaemia and improved quality of sleep in multiple studies [39–42]. In addition to survey data, qualitative data from parental interviews is overwhelmingly positive, highlighting improvements in parental mental health and quality of life, as well as increased parental confidence in outsourcing childcare, with AID use in the child [40, 43, 44].

Children and adolescents with type 1 diabetes generally self-report high satisfaction and acceptability of AID, although evidence of statistically significant improvements in other child-reported psychosocial metrics is harder to find [45, 46]. Other studies have shown clear benefits in child-reported metrics including decreased fear of hypoglycaemia, decreased diabetes distress and increased well-being [47]. Qualitative data from interviews of children and adolescents on AID have also been positive, with children reporting feeling more rested and less worried about their illness after starting AID [43, 44, 47].

## Application of technology in real life and practical considerations

Early commercially available AID systems were found to have a high rate of discontinuation and suboptimal time in closed loop when used by children with type 1 diabetes [48]. However, AID usability has improved over time. Numerous recent publications of real-world data show that children with type 1 diabetes are able to achieve a consistently high rate of time in closed loop and achieve glycaemic benefits similar to those observed in RCTs across all AID systems [17, 49–52]. The rate of AID discontinuation reported in these real-world studies is reassuringly low.

It is important for the clinical team to set realistic expectations and appropriately counsel families at the time of starting any new technology [53]. CGM readings do not always perfectly match glucometer readings, particularly when glucose is changing rapidly. This can be confusing and frustrating for users if they are not appropriately counselled on what to expect and also creates distrust in the technology. Users of technology need to learn how to troubleshoot data gaps and connectivity issues. Additionally, devices do not always stay on the body for the approved number of days and overlay patches and extra skin preparations are often necessary. Children often report pain with device insertion and skin irritation from the adhesive is common, at times limiting usability [54]. Like in open-loop pumps, infusion-set failure remains common in children using AID and can potentially lead to diabetic ketoacidosis (DKA) if not addressed promptly. Importantly, fewer DKA episodes are observed in children using diabetes technology as compared with those who are not [8].

There are a number of paediatric-specific practical issues with technology use, including the need to train school nurses and other caregivers on how to use a new device and respond to alarms. Remote data sharing has certainly improved safety, although parents are often now more involved than ever in the child’s daily diabetes management at school and communicate with the nurse or teacher multiple times per day about their child’s glucose trends, mealtime dosing and management of exercise during the school day [55].

A final consideration is the timing of technology introduction following diagnosis. CGM introduction now occurs quite early for many children with type 1 diabetes, with documented benefits to early use. However, there tends to be a lag in AID introduction for even the most motivated families, largely due in the USA to insurance constraints. Most of the pivotal AID studies excluded children who were diagnosed <6 months prior to study enrolment to avoid overlap with the ‘honeymoon period’ when glycaemic management almost universally improves as endogenous insulin production transiently resumes. Clinicians have often been reluctant to prescribe AID right at the time of diagnosis, largely related to concerns regarding the ability of a family to learn the complexities of AID in addition to the extensive diabetes teaching necessary at the time of diagnosis. However, there are now two large RCTs that have clearly documented successful early AID introduction. Although these studies do not show that beta cell function is preserved with early AID use, they do suggest that early AID introduction is likely safe and clearly effective at improving glycaemic outcomes following diagnosis [56, 57]. Moreover, evidence suggests that early AID use might diminish the negative glycaemic impact of DKA at the time of diagnosis [58]. As such, it is likely that the gap between diabetes diagnosis and AID introduction will continue to shorten over time.

## Barriers to technology use

Multiple barriers exist that limit more widespread use of diabetes technology in children. Adolescents and even younger children may be resistant to having a physical device attached to them [59] and young children have a limited body surface area on which to place devices [54, 60]. Although most families are reassured by data, others feel more anxious about diabetes when monitoring real-time CGM data [54]. Similarly, children and adolescents commonly eschew attracting attention to themselves, due to cosmetic issues or the embarrassment of highlighting their diabetes status in front of classmates [61, 62]. The audible alarms for high or low glucose levels are a frequent source of frustration that cause some to discontinue use [63].

When a child resists wearing a pump but is willing to use a CGM, smart insulin pens connected via Bluetooth to phone apps can provide an alternative option to benefit further from technology. Potential benefits of smart pens include insulin dose calculation with adjustment for insulin on board, the ability to set reminders and the generation of data to aid clinicians in making dose adjustments. In a recent small RCT, initiation of a smart pen cap in adolescents and adults with type 1 diabetes was associated with a 5.2% increase in TIR [64]. An earlier observational study reported a reduction in hypoglycaemia but not hyperglycaemia in children with type 1 diabetes after smart pen introduction [65]. More high-quality evidence is needed to determine the impact of smart pen use in children with type 1 diabetes.

The high cost of diabetes technology is also a barrier to use and global coverage by many national healthcare systems remains limited [66]. There has been an increase in coverage in many countries over time. In Europe, and even more so in the USA, there have been gaps in coverage of technology use in families of lower socioeconomic status (SES) [67]. Importantly, a recent study evaluating predictors of success with AID use did not find a difference when comparing US families receiving Medicaid with those having private insurance, suggesting that all children benefit from AID use regardless of SES [68]. Furthermore, recent data suggest that AID systems are actually cost effective compared with standard care due to improved glycaemic outcomes [69, 70].

Healthcare providers can also act as a barrier to technology access, sometimes serving as ‘gatekeepers’ by only offering options to families they feel can handle the increased complexity of technology-based tools [71, 72]. There appear to be racial/ethnic differences in technology use that exist beyond differences in SES. In one study of CGM use among US families with commercial insurance in the first year following type 1 diabetes diagnosis, 96% of non-Hispanic white children vs 73% of black children were using CGM [73].

Given that AID trials have found that the greatest magnitude of benefit occurs in children and adolescents with the lowest baseline TIR [32], there are growing arguments to expand access among all groups, including minority populations that have historically exhibited low rates of technology use [71, 74, 75]. Further, the research performed in this area has over-represented individuals of higher SES, and there are clear needs to learn the predictors of success among under-represented groups to better identify approaches that are likely to be efficacious among all groups [76].

## Future research

Future research in this area will focus not only on continued improvement in glycaemic outcomes but also on improvement in patients’ quality of life. A goal of many future systems is to function without user input, referred to as fully closed-loop (FCL) systems. Due to the experimental nature of these, most have been tested predominantly in adults. FCL systems use additional features for detecting food intake that do not require user action (e.g. a smart watch that detects eating and drinking movements, algorithmic assessments of CGM flux likely associated with a meal, system anticipation of meals or more-aggressive insulin administration coupled with glucagon injection) [77–80]. Other approaches have focused on accounting for exercise, either via anticipation or heart-rate information [81, 82].

Head-to-head trials comparing systems used with and without carbohydrate announcement demonstrate that announcing carbohydrate intake still results in greater TIR; however, studies also show that systems function better than existing systems when meals are not announced [78]. Trials have demonstrated in many cases acceptable TIR when using AID in FCL (58–78%), which could improve glycaemic management in children and adolescents who forget or choose not to bolus for meals [78, 80, 83].

Another area where AID systems could be improved is in reducing the delay between sensing an elevation in glucose and the action of the injected insulins. Thus, faster-acting insulins have been a source of hope for improving postprandial glycaemic management. Some studies have suggested minor glycaemic improvements are obtained with use of an ultra-rapid-acting insulin with AID, although no glycaemic improvements have been found in paediatric studies [84, 85]. Some published work indicates that reworking of the controller algorithm or the insulin settings may be necessary to achieve more complete efficacy [86–88].

Other approaches tested in adults have focused on adding other diabetes medications to AID use, including pramlintide (which increased TIR by 10% when co-secreted with insulin) [89], semaglutide (which reduced insulin requirements in early type 1 diabetes) [90] and sodium–glucose cotransporter 2 (SGLT2) inhibitors (which increased TIR by 9.9% when taken orally alongside AID use) [91]. Still, with SGLT2s, the potential for unrecognised ketosis likely limits this use in adolescents until continuous ketone monitoring becomes available [92]. It is still unclear whether the use of automated glucagon delivery with an AID system will greatly improve care, as comparisons of TIR between insulin alone or bihormonal systems have been complicated by trial design and optimised systems have not been tested head-to-head [93]. A recent prospective study of an FCL bihormonal system in adults reported impressive glycaemic outcomes (TIR 80.3%), although there was a fairly high dropout rate largely due to the burden required to use the system (two CGMs and two pumps/infusion sets are required) [94].

## New horizons

Compared with previous paradigm-shifting approaches, the process towards development, approval and widespread commercial availability of AID systems has progressed rapidly. Still, more rapid advancement may be allowed if there is greater ability for users to switch between individual components. This shift began in 2023 and now multiple AID systems pair with two or more CGMs. This interoperability between brands will allow users to benefit from ongoing improvements between one CGM type and another and allow individuals to select CGM and AID devices that best suit their needs.

Outside of AID control, there has been interest in using predictive algorithms to provide automated advice to patients or physicians. These decision-support systems have met with limited success thus far [95–97], highlighting the complexities of an approach that highly depends on reliable data over time and the user’s willingness to follow through with recommendations [98]. Still, this type of approach should eventually help in optimising insulin dosing variables.

Finally, with respect to the inequalities of use of diabetes technology among under-represented populations, there is a need for research that could lead to these technologies becoming more accessible and available [76]. This includes demonstrations of ease-of-use and efficacy in all populations to convince all levels of decision makers about the need to increase access to all people with diabetes [75].

## Conclusions

It is an exciting time for paediatric endocrinologists, diabetes researchers and children with type 1 diabetes and their families as advances in diabetes technology continue to be made. Reliable CGMs opened the door to successful AID development, which in turn has improved glycaemic outcomes and decreased the burden of diabetes for children with type 1 diabetes and their families. Affordability and access for all to technology should be a top priority moving forward. Although the impact of AID use on long-term diabetes complications remains to be determined, a reduction in complications is certainly to be expected given the positive impact this technology has had on glycaemic management in youth with type 1 diabetes.

## Abbreviations

AID: Automated insulin delivery
CGM: Continuous glucose monitor
DKA: Diabetic ketoacidosis
FCL: Fully closed-loop
MDI: Multiple daily insulin injections
SES: Socioeconomic status
SGLT2: Sodium–glucose cotransporter 2
TIR: Time in range
